# Effects of Overexpression of Neurosecretory Protein GL-Precursor Gene on Glucose Homeostasis and Insulin Sensitivity in Mice

**DOI:** 10.3390/ijms22094681

**Published:** 2021-04-28

**Authors:** Keisuke Fukumura, Yuki Narimatsu, Shogo Moriwaki, Eiko Iwakoshi-Ukena, Megumi Furumitsu, Kazuyoshi Ukena

**Affiliations:** Laboratory of Neurometabolism, Graduate School of Integrated Sciences for Life, Hiroshima University, Higashi-Hiroshima, Hiroshima 739-8521, Japan; kfuku@hiroshima-u.ac.jp (K.F.); d214243@hiroshima-u.ac.jp (Y.N.); m203300@hiroshima-u.ac.jp (S.M.); iwakoshi@hiroshima-u.ac.jp (E.I.-U.); mfurumi@hiroshima-u.ac.jp (M.F.)

**Keywords:** neurosecretory protein GL, hypothalamus, neuropeptide, obesity, glucose homeostasis, insulin sensitivity

## Abstract

A high-fat diet (HFD) quickly induces obesity with insulin resistance and hyperglycemia. We previously reported that a novel hypothalamic small protein, named neurosecretory protein GL (NPGL), stimulates feeding and fat accumulation in mice. However, the effects of NPGL on insulin sensitivity and glucose homeostasis remain unknown. Hence, we subjected NPGL-precursor gene (*Npgl*)-overexpressing mice to the oral glucose tolerance test (OGTT) and intraperitoneal insulin tolerance test (IPITT) under normal chow (NC) and HFD conditions. *Npgl* overexpression promoted body mass gain and tended to increase food intake of NC-fed mice, whereas it had little effect on HFD-fed mice. The OGTT showed elevated blood glucose and insulin levels in *Npgl*-overexpressing NC-fed mice 15 min after glucose administration. Both the OGTT and IPITT demonstrated that *Npgl* overexpression decreased blood glucose levels in HFD-fed mice 60 min after glucose and insulin treatments. Notably, *Npgl* overexpression increased adipose tissue masses only in NC-fed mice, and it decreased blood glucose and insulin levels in HFD-fed mice at the experimental end point. It also increased the mRNA expression of galanin, one of the feeding and metabolic regulatory neuropeptides, in the hypothalamus of HFD-fed mice. Therefore, NPGL may alleviate HFD-induced hyperglycemia and insulin resistance in mice.

## 1. Introduction

Obesity is a disease that has reached epidemic proportions. It is a major health-related concern worldwide because this condition and its comorbidities, such as depression, type 2 diabetes, cardiovascular disease, and certain cancers, have created a huge economic burden [1,2,3]. The increase in the prevalence of obesity has emphasized the need for research focusing on its biological causative factors [3]. During obesity development, excess fat accumulation promotes insulin resistance and glucose intolerance, which is generally accompanied by chronic inflammation in adipose tissue [4,5,6]. As overfeeding and/or biased feeding, such as continuous feeding of a high-fat diet (HFD), quickly leads to metabolic disorders, the regulatory mechanisms of feeding behavior and metabolism have been investigated [7,8,9]. To date, several hypothalamic neuropeptides involved in feeding behavior have been identified in the arcuate nucleus of the hypothalamus, for instance, potent orexigenic factors such as neuropeptide Y (NPY) and agouti-related peptide (AgRP), and the anorexigenic factor, proopiomelanocortin (POMC)-derived α-melanocyte-stimulating hormone [7,8,9]. Among peripheral factors, ghrelin and leptin are well-known, feeding-regulatory peptides. Ghrelin, an orexigenic peptide secreted by the stomach, stimulates feeding behavior via NPY/AgRP neurons [10,11,12]. Leptin, an anorexigenic polypeptide secreted from white adipose tissue (WAT) in proportion to body fat reserves, influences NPY/AgRP and POMC neuron activities [13,14,15,16]. Moreover, insulin, a key regulator secreted from pancreatic β-cells, converts dietary carbohydrates into fat deposits and maintains systemic glucose homeostasis [17,18]. Although many factors involved in the regulation of energy homeostasis have been identified in the last few decades, the hormonal controls of insulin sensitivity and glucose homeostasis that underlie obesity development are not fully understood.

To elucidate the regulatory mechanism of energy homeostasis, we investigated previously unknown bioactive substances and their modes of action in animals. The search for novel neuropeptides and peptide hormone precursors in the hypothalamus led to the identification of a novel cDNA in the chick hypothalamus [19]. As the deduced precursor protein contains a small secretory protein of 80 amino acids with Gly-Leu-NH_2_ at the C-terminus, the novel neuropeptide has been named neurosecretory protein GL (NPGL) [19]. Furthermore, homologous NPGL proteins have been discovered in mammals, including humans, rats, and mice, suggesting that the primary structure of NPGL is highly conserved in mammals and avian species [20]. In addition, its paralogous neuropeptide has been identified and named neurosecretory protein GM [21]. Similar to the effects of NPGL infusion on energy metabolism in avian species [22,23], acute intracerebroventricular (i.c.v.) infusion of NPGL stimulates feeding behavior in mice fed with normal chow (NC) [24]. Chronic i.c.v. infusion of NPGL decreases locomotor activity during the dark period in mice [25]. In addition, chronic i.c.v. infusion of NPGL increases food intake with considerable fat accumulation in mice fed with a medium fat/medium sucrose diet (MFSD), whereas it induces moderate fat accumulation without changing food intake in NC-fed mice [25]. We previously showed that chronic i.c.v. infusion of NPGL elicits food intake and subsequent fat accumulation through de novo lipogenesis in rats [26]. Notably, overexpression of the NPGL-precursor gene (*Npgl*) in the mouse hypothalamus increases food intake and fat deposits under NC and MFSD conditions and elevates blood insulin levels without changing the blood glucose levels [27]. However, the effects of NPGL on insulin sensitivity and glucose homeostasis have not been elucidated.

In this study, we subjected *Npgl*-overexpressing mice to the oral glucose tolerance test (OGTT) and intraperitoneal insulin tolerance test (IPITT) under a series of NC and HFD feedings to investigate whether NPGL affects insulin sensitivity and glucose tolerance. Additionally, we measured body mass gain, food intake, body composition, blood parameters, and mRNA expression of feeding and metabolic regulatory genes at the experimental end point to determine the effects of HFD feeding on NPGL action in mice.

## 2. Results

### 2.1. Effects of NPGL-Precursor Gene Overexpression on Food Intake and Body Mass Gain under NC and HFD Conditions

**Figure 1:**
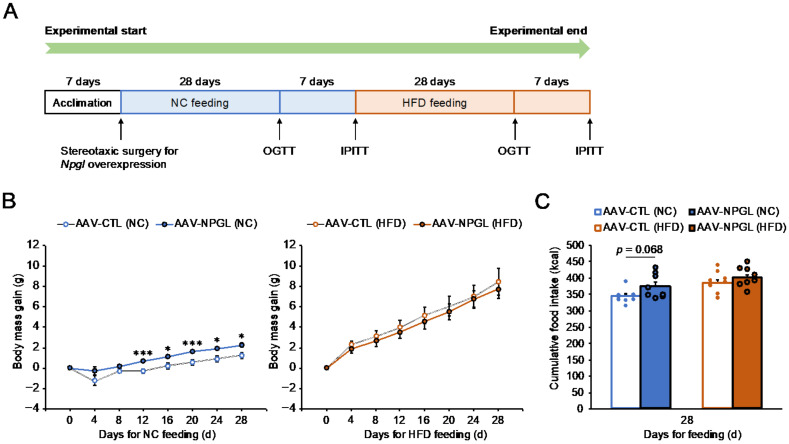
Effects of *Npgl* overexpression on body mass gain and food intake in normal chow (NC)-fed and high-fat diet (HFD)-fed mice. The panels show the data obtained upon injection of the AAV-based control vector (AAV-CTL) or the AAV-based NPGL-precursor gene vector (AAV-NPGL) in NC-fed and HFD-fed mice. (**A**) Experimental procedure. After animal surgery, mice were fed NC for 28 days until the first oral glucose tolerance test (OGTT) and intraperitoneal insulin tolerance test (IPITT). Thereafter, the diet was changed to an HFD and mice were fed for 28 days until the second OGTT and IPITT. (**B**) Body mass gain and (**C**) cumulative food intake. Circles in **C** represent individual data points. Each value represents the mean ± standard error of the mean (*n* = 8; * *p* < 0.05, *** *p* < 0.005 for Student’s *t*-test).

To determine the effects of NPGL on feeding behavior, metabolism, glucose homeostasis, and insulin sensitivity under NC and HFD conditions, we conducted a series of experiments, as shown in Figure 1A. Briefly, mice were fed NC for 28 days for the first OGTT and IPITT, and subsequently fed an HFD for 28 days for the second OGTT and IPITT. When NC feeding was started, control mice and *Npgl*-overexpressing mice were weighed at 23.6 ± 0.2 g and 23.4 ± 0.3 g, respectively. When subsequent HFD feeding was started, control mice and *Npgl*-overexpressing mice were weighed at 25.6 ± 0.4 g and 26.7 ± 0.3 g, respectively. Quantitative RT-PCR (qRT-PCR) showed chronic adeno-associated virus (AAV)-induced *Npgl* overexpression in the mediobasal hypothalamus (MBH) of mice at the experimental end point (Figure S1). *Npgl* overexpression significantly increased body mass gain from day 12 and tended to increase cumulative food intake in mice fed with NC for 28 days (Figure 1B,C). In contrast, *Npgl* overexpression did not affect body mass gain or cumulative food intake under HFD conditions (Figure 1B,C).

### 2.2. Effects of NPGL-Precursor Gene Overexpression on Glucose Homeostasis and Insulin Sensitivity under NC Conditions

**Figure 2:**
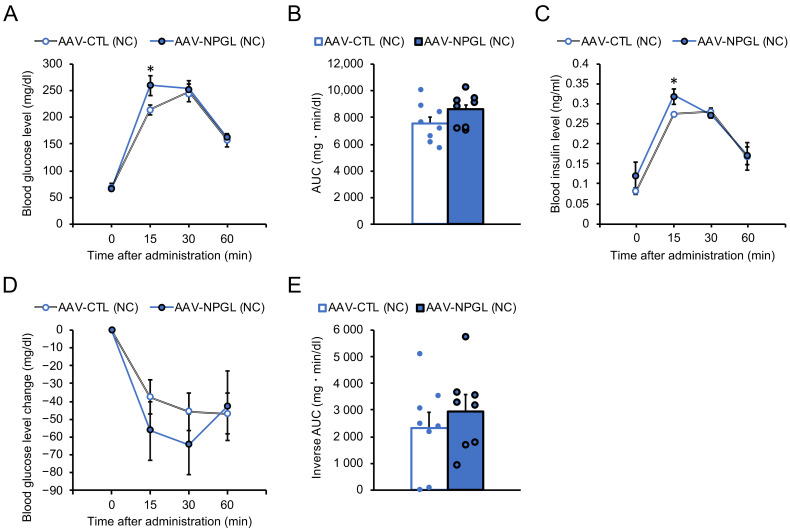
Effects of *Npgl* overexpression on glucose tolerance and insulin sensitivity in normal chow (NC)-fed mice. The panels show the data obtained upon injection of the AAV-based control vector (AAV-CTL) or the AAV-based NPGL-precursor gene vector (AAV-NPGL) in NC-fed mice. (**A**–**C**) Results of oral glucose tolerance test (OGTT) (**A**: blood glucose levels, **B**: area under the curve (AUC) for blood glucose levels, **C**: corresponding blood insulin secretion curves) for NC-fed mice at multiple time points. (**D**,**E**) Results of intraperitoneal insulin tolerance test (IPITT) (**D**: changes in blood glucose levels compared with those at time 0, **E**: inverse AUC for blood glucose levels) for NC-fed mice at multiple time points. Circles in **B** and **E** represent individual data points. Each value represents the mean ± standard error of the mean (*n* = 8; * *p* < 0.05 for Student’s *t*-test).

After 28 days of *Npgl* overexpression under NC conditions, we performed the OGTT. Blood glucose and insulin levels were significantly higher 15 min after oral glucose administration in *Npgl*-overexpressing mice (Figure 2A,C). In contrast, a calculation of the area under the curve (AUC) above the glucose baseline showed no significant difference between control and *Npgl*-overexpressing mice (Figure 2B). After one week of recovery from OGTT damage, we performed the IPITT. There was no significant difference in the blood glucose level and inverse AUC below the glucose baseline after intraperitoneal insulin administration (Figure 2D,E).

### 2.3. Effects of NPGL-Precursor Gene Overexpression on Glucose Homeostasis and Insulin Sensitivity under HFD Conditions

**Figure 3:**
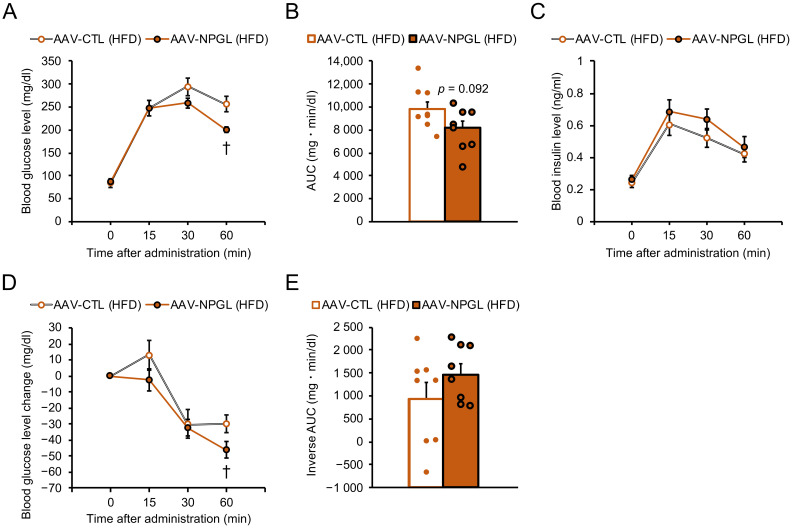
Effects of *Npgl* overexpression on glucose tolerance and insulin sensitivity in high-fat diet (HFD)-fed mice. The panels show the data obtained upon injection of the AAV-based control vector (AAV-CTL) or the AAV-based NPGL-precursor gene vector (AAV-NPGL) in HFD-fed mice. (**A**–**C**) Results of oral glucose tolerance test (OGTT) ((**A**): blood glucose levels, (**B**): area under the curve (AUC) for blood glucose levels, (**C**): corresponding blood insulin secretion curves) for HFD-fed mice at multiple time points. (**D**,**E**) Results of intraperitoneal insulin tolerance test (IPITT) ((**D**): changes in blood glucose levels compared with those at time 0, (**E**): inverse AUC for blood glucose levels) for HFD-fed mice at multiple time points. Circles in (**B**,**E**) represent individual data points. Each value represents the mean ± standard error of the mean (*n* = 8; † *p* < 0.05 for Student’s *t*-test).

After the OGTT and IPITT under NC conditions, we changed the diet from NC to an HFD and performed the same tests after 28 days of rearing under HFD conditions (Figure 1A). In the OGTT, the blood glucose level was significantly lower 60 min after oral glucose administration in *Npgl*-overexpressing mice (Figure 3A). In addition, a slight decrease in the AUC was observed for *Npgl*-overexpressing mice (Figure 3B), whereas there was no significant difference in the blood insulin levels (Figure 3C). After one week of recovery from OGTT damage, we performed the IPITT. In *Npgl*-overexpressing mice, the blood glucose level was significantly lower 60 min after intraperitoneal insulin administration, and the inverse AUC below the glucose baseline tended to be higher but without a significant difference (Figure 3D,E).

### 2.4. Effects of NPGL-Precursor Gene Overexpression on Body Composition and Blood Parameters

**Figure 4:**
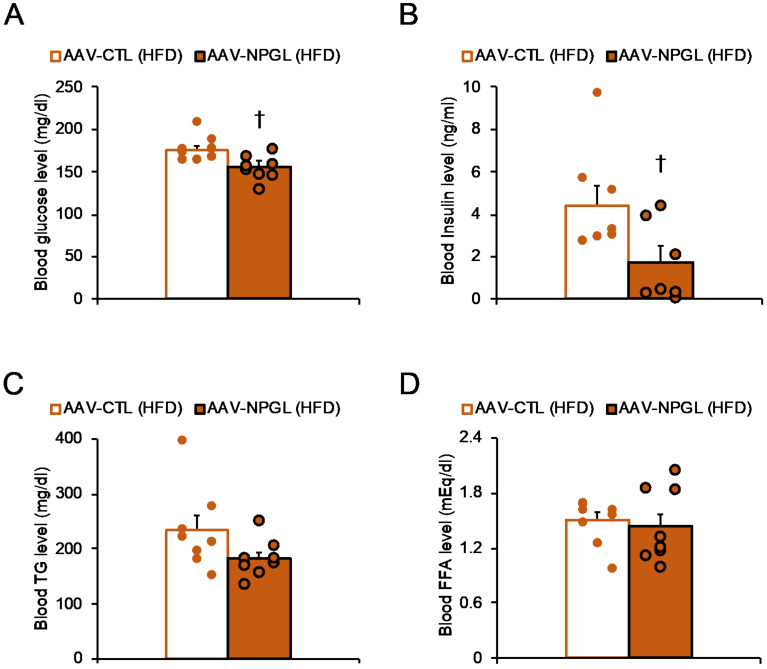
Effects of *Npgl* overexpression on blood parameters in high-fat diet (HFD)-fed mice. The panels show the data obtained upon injection of the AAV-based control vector (AAV-CTL) or the AAV-based NPGL-precursor gene vector (AAV-NPGL) in HFD-fed mice. (**A**) Blood glucose, (**B**) insulin, (**C**) triglyceride (TG), and (**D**) free fatty acid (FFA) levels. Circles represent individual data points. Each value represents the mean ± standard error of the mean ((**A**,**C**,**D**), *n* = 8; **B**, *n* = 7; † *p* < 0.05 for Student’s *t*-test).

To examine the effects of nutrition on NPGL action with respect to body composition and blood parameters, we measured the masses of adipose tissues, muscle, and several organs under NC and HFD conditions. Independent of the series of experiments described in Figure 1A, we overexpressed *Npgl* in the hypothalamus for 40 days to analyze body composition under NC conditions. Under NC, the masses of interscapular brown adipose tissue (BAT) and WAT were significantly higher in *Npgl*-overexpressing mice than in control mice (Figure S2A,B). In contrast, there were no significant differences in the tissue masses of the control and *Npgl*-overexpressing mice under HFD conditions (Figure S2A,B). In addition, under both feeding conditions, *Npgl* overexpression did not affect the masses of the gastrocnemius muscle, liver, testis, kidney, and heart (Figure S3). Notably, the blood glucose and insulin levels at the experimental end point without fasting under HFD conditions were significantly lower in *Npgl*-overexpressing mice, whereas there were no changes in blood triglyceride (TG) and free fatty acid (FFA) levels (Figure 4).

### 2.5. Effects of NPGL-Precursor Gene Overexpression on mRNA Expression of Neuropeptides and Genes Related to Gluconeogenesis and Glucose Uptake

**Figure 5:**
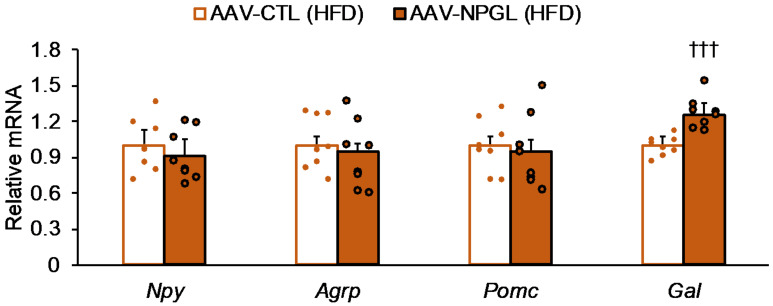
Effects of *Npgl* overexpression on mRNA expression of neuropeptides in high-fat diet (HFD)-fed mice. The graph shows the data obtained upon injection of the AAV-based control vector (AAV-CTL) or the AAV-based NPGL-precursor gene vector (AAV-NPGL) in HFD-fed mice. The mRNA expression levels of neuropeptide Y (*Npy*), agouti-related peptide (*Agrp*), proopiomelanocortin (*Pomc*), and galanin (*Gal*) in the mediobasal hypothalamus. Circles represent individual data points. Each value represents the mean ± standard error of the mean (*n* = 8; ††† *p* < 0.005 for Student’s *t*-test).

Since we observed decreases in blood glucose levels both in the OGTT and IPITT under HFD conditions and at the end point of a series of experiments, we measured the mRNA expression levels of neuropeptides, which are involved in whole-body energy metabolism, in the MBH. The qRT-PCR showed that *Npgl* overexpression increased the mRNA expression of galanin (*Gal*), whereas it had no effect on the expression of *Npy*, *Agrp*, or *Pomc* under HFD conditions (Figure 5). On the other hand, we measured mRNA expression of genes related to lipid metabolism, glycolysis, glucose and lipid uptake, and browning in the inguinal WAT (iWAT), such as acetyl-CoA carboxylase (*Acc*), fatty acid synthase (*Fas*), carbohydrate-responsive element-binding protein α (*Chrebpα*), carnitine palmitoyltransferase 1a (*Cpt1a*), adipose triglyceride lipase (*Atgl*), hormone-sensitive lipase (*Hsl*), glyceraldehyde-3-phosphate dehydrogenase (*Gapdh*), solute carrier family 2 member 4 (*Slc2a4*), cluster of differentiation 36 (*Cd36*), peroxisome proliferator-activated receptor γ coactivator 1α (*Pgc1α*), uncoupling protein 1 (*Ucp1*), and type II iodothyronine deiodinase (*Dio2*). However, *Npgl* overexpression did not affect the expression of these genes (Figure S4). In addition, when we measured mRNA expression of genes related to gluconeogenesis and glucose uptake in the liver, such as glucose-6-phosphatase (*G6pase*), phosphoenolpyruvate carboxykinase (*Pepck*), solute carrier family 2 member 2 (*Slc2a2*), and fibroblast growth factor 21 (*Fgf21*), *Npgl* overexpression induced no changes in these genes (Figure S5).

## 3. Discussion

Hypothalamic neuropeptides predominantly control feeding behavior and metabolism and are closely linked to obesity development [28,29]. We recently demonstrated that hypothalamic overexpression of *Npgl*, a novel small secretory protein precursor gene, elicits food intake and fat accumulation in mice [27]. However, the effect of NPGL on insulin sensitivity and glucose homeostasis has not been elucidated. In this study, we subjected *Npgl*-overexpressing mice to the OGTT and IPITT under both NC and HFD conditions. Our data showed that *Npgl* overexpression under HFD conditions restrained an increase in blood glucose level in the OGTT, whereas it promoted a decrease in blood glucose level in the IPITT. Furthermore, *Npgl* overexpression reduced blood glucose and insulin levels in HFD-fed mice at the experimental end point.

Our data highlighted the effects of *Npgl* overexpression, which mitigated glucose intolerance in the OGTT and insulin resistance in the IPITT under HFD conditions. In contrast, we observed increases in blood glucose and insulin levels 15 min after oral glucose administration in *Npgl*-overexpressing mice under NC conditions. Several studies have indicated that an increase in blood glucose level immediately after glucose administration can reflect glucose absorption into the circulation through the intestine [30,31,32]. In addition, our previous study has suggested that *Npgl* overexpression promotes the absorption of glucose, as a substrate of de novo lipogenesis, from the circulation into WAT in NC-fed mice [27]. These data imply that NPGL affects multiple tissues, such as the intestine and WAT, to orchestrate glucose absorption and carbohydrate use for efficient de novo lipogenesis under NC conditions. In contrast to our previous study demonstrating that *Npgl* overexpression is unable to affect the blood glucose level in NC-fed mice [27], we again emphasize the finding that *Npgl* overexpression alleviated glucose intolerance, insulin resistance, and hyperglycemia in HFD-fed mice at the experimental end point of the present study. Over the past 30 years, novel molecular mechanisms linking obesity and its related disorders have been deciphered. Although the field has primarily focused on the direct impact of obesity-associated alterations in peripheral tissues such as the liver, skeletal muscle, and adipose tissue, the role of the central nervous system as a regulator of energy homeostasis among different organs has not received the same attention [33]. In this study, we found that *Npgl* overexpression upregulated the mRNA expression of *Gal*, a 29-amino-acid peptide, in the hypothalamus. Several studies have recently uncovered new aspects of neuropeptides in energy metabolism. For instance, GAL ameliorates insulin resistance and improves glucose metabolism by activating the trafficking of glucose transporter 4 and glucose uptake in the skeletal muscle and adipose tissue [34]. In addition, we have shown the co-localization of GAL and NPGL in the same neurons of the hypothalamic arcuate nucleus in mice [25], raising the possibility that these neuropeptides modulate each other at the transcriptional level, perhaps via the autocrine system. In contrast, the receptor for NPGL and its intracellular signaling remain unidentified. Although our data are limited to transcriptional changes of the neuropeptides, future studies to analyze central hormonal relay and peripheral insulin signaling will open up new avenues for the hypothalamic regulation of insulin sensitivity and glucose homeostasis.

In this study, we observed differences in the effects of *Npgl* overexpression on body mass gain and fat accumulation under NC and HFD conditions. *Npgl* overexpression increased adipose tissue mass under NC conditions, whereas it had little effect under HFD conditions. We previously demonstrated that NPGL stimulates fat accumulation in WAT through de novo lipogenesis using dietary carbohydrates in rats [26]. In addition, we recently revealed that NPGL promotes fat accumulation in high-sucrose diet (HSD)-fed rats, although it does not induce an increase in food intake [35]. In contrast, it is well known that an HFD suppresses de novo lipogenesis in rodents [36,37]. Moreover, the present data indicated that *Npgl* overexpression hardly increased WAT mass in HFD-fed mice. In addition, we could not observe activated de novo lipogenesis at the transcriptional level in the WAT of *Npgl*-overexpressing HFD-fed mice (data not shown). Dietary carbohydrates and fat regulate de novo lipogenesis partially via certain transcriptional factors in adipose tissue. Among them, carbohydrate response element binding protein (ChREBP) is a critical transcriptional factor of systemic lipid metabolism, including de novo lipogenesis, in various peripheral tissues [38]. ChREBP is activated and promotes de novo lipogenesis in response to carbohydrate intake, whereas dietary fat, such as polyunsaturated fatty acids, inhibits ChREBP-induced de novo lipogenesis [39]. It has been shown that *Npgl* overexpression upregulates mRNA expression of *ChREBP* in mice fed with NC and an MFSD, which include a large amount of carbohydrates [27]. Hence, we speculate that dietary carbohydrates and fat have opposite effects on NPGL action in fat accumulation, perhaps via the transcriptional factors in peripheral tissues, such as WAT. Since these transcriptional factors are regulated by post-translational modification as well as at the transcriptional and translational levels, detailed analysis is required to understand the regulatory mechanisms of lipid metabolism by NPGL. On the other hand, BAT mass was also increased in NC-fed mice, but not HFD-fed mice in the present study. However, the regulatory mechanisms of fat accumulation in the BAT and the functional relationship between the WAT and BAT remain unknown at this time.

Our data supported the finding that *Npgl* overexpression stimulates food intake in NC-fed mice [27], whereas it did not affect feeding behavior under HFD conditions. To date, a considerable amount of research has revealed that dietary nutrients and metabolic status influence neuropeptide functions in feeding behavior and metabolism [40,41]. For instance, fasting and long-term HFD feeding evoke neuronal activation, including increased spike frequency in NPY/AgRP neurons via peripheral signaling in mice [42,43]. Similarly, we demonstrated that NPGL stimulates food intake at different intensities under feeding with different nutritional compositions, based on the species. Under MFSD conditions, NPGL significantly stimulates food intake, whereas it has little effect under NC conditions in rats [26]. In addition, we recently showed that NPGL cannot induce an increase in food intake of an HSD in rats [35]. Since both HFD and HSD are highly unbalanced nutrient diets, NPGL might stimulate feeding behavior only toward MFSD. A recent report demonstrated that the hypothalamic corticotropin-releasing hormone promotes the intake of carbohydrate over fat in mice [44]. Peripheral FGF21 suppresses simple sugar intake as a negative feedback in response to dietary carbohydrates in mice [45,46]. In contrast, although we observed that NPGL-like immunoreactive fibers contact the anorexigenic POMC neurons in the arcuate nucleus in mice [24], the molecular mechanisms by which NPGL influences feeding behavior remain unclear. Therefore, further study is needed to elucidate the molecular mechanisms of feeding behavior, including feeding preferences.

In summary, this study revealed that *Npgl* overexpression exerted different effects on feeding behavior and fat accumulation under NC and HFD conditions. The results of a series of previous studies [25,26,27] strongly suggest that NPGL plays multiple roles in energy homeostasis according to dietary nutrients. The present data showing the differences of NPGL action in body mass gain and fat accumulation under NC and HFD conditions require future study to analyze other metabolic parameters, including locomotor activity and energy expenditure under different nutrition. Notably, under HFD conditions, the OGTT and IPITT revealed a novel function of NPGL associated with insulin sensitivity and glucose homeostasis. Based on the reduced blood glucose and insulin levels in *Npgl*-overexpressing HFD-fed mice at the experimental end point, we propose that NPGL prevents HFD-induced glucose intolerance, insulin resistance, and hyperglycemia in mice. Further research on the NPGL action, including analysis of loss of function as well as *Npgl* overexpression, will help understand the complicated mechanisms of the central and peripheral energy metabolisms, including nutrient selection, obesity development, and related disorders.

## 4. Material and Methods

### 4.1. Animals

Male C57BL/6J mice (7 weeks old) were purchased from SLC (Hamamatsu, Japan) and housed individually in general cages (l: 26 cm, w: 18 cm, h: 13 cm, CL-0103-2; CLEA Japan, Tokyo, Japan) under standard conditions (25 ± 1 °C under a 12-h light/dark cycle) with ad libitum access to water and NC (CE-2; CLEA Japan) until animal surgery for *Npgl* overexpression. Thereafter, the mice were fed NC followed by an HFD (45% of calories from fat/17.5% of calories from sucrose, D12451; Research Diets, New Brunswick, NJ, USA), as described below. Animals were operated on under isoflurane anesthesia.

### 4.2. Production of AAV-Based Vectors

AAV-based vectors were produced following a previously reported method [26]. In the present study, the primers for mouse *Npgl* were 5′-CGATCGATACCATGGCTGATCCTGGGC-3′ (sense primer) and 5′-CGGAATTCTTATTTTCTCTTTACTTCCAGC-3′ (antisense primer). The AAV-based vectors were prepared at a concentration of 1 × 10^9^ particles/µL and stored at −80 °C until use.

### 4.3. Npgl Overexpression

For *Npgl* overexpression, mice were bilaterally injected with 0.5 µL/site (5.0 × 10^8^ particles/site) of AAV-based vectors (AAV-NPGL or AAV-CTL), using a Neuros Syringe (7001 KH; Hamilton, Reno, NV, USA), at the mediobasal hypothalamic region with the following coordinates: 2.2 mm caudal to the bregma, 0.25 mm lateral to the midline, and 5.8 mm ventral to the skull surface. *Npgl* overexpression was maintained for 70 days during a series of experiments, as shown in Figure 1A, or for 40 days in mice fed with NC alone. *Npgl* overexpression was confirmed by qRT-PCR at the experimental end point. Food intake and body mass were measured every morning (9:00 a.m.). Body composition and blood parameters were measured at the experimental end point of *Npgl* overexpression.

### 4.4. OGTT and IPITT

The OGTT and IPITT were performed according to a previously reported method [47]. Briefly, mice were fasted for 16 h (overnight fasting) for the OGTT and 4 h (morning fasting) for the IPITT at weekly intervals. Using the GLUCOCARD G+ (Arkray, Kyoto, Japan), blood glucose levels were measured 0, 15, 30, and 60 min after oral glucose (1 g/kg body weight) administration and intraperitoneal insulin (0.75 units/kg) injection. A 35-µL blood sample was collected from the tail vein using a heparinized plastic hematocrit tube (Drummond Scientific Company, Broomall, PA, USA), and plasma was separated by centrifugation at 2500× *g* for 30 min. After centrifugation, the plasma was stored at −80 °C for future insulin measurement. The Rebis Insulin-mouse U ELISA kit (Shibayagi, Gunma, Japan) was used to measure insulin levels. The AUC for blood glucose was calculated using the linear trapezoidal method for both the OGTT and IPITT.

### 4.5. Quantitative RT-PCR

The MBH was dissected using fine forceps and small scissors, according to the mouse brain atlas [48], and snap frozen in liquid nitrogen for RNA processing. The regions included the supraoptic nucleus, dorsomedial hypothalamus, ventromedial hypothalamus, arcuate nucleus, lateral hypothalamic area, and mammillary nucleus. Total RNA was extracted using TRIzol reagent (Invitrogen, Carlsbad, CA, USA) for the MBH and liver, and QIAzol lysis reagent (QIAGEN, Venlo, Netherlands) for the iWAT in accordance with the manufacturer’s instructions. First-strand cDNA was synthesized from total RNA using a PrimeScript RT reagent Kit with gDNA Eraser (Takara Bio, Shiga, Japan).

The primer sequences used in this study are listed in Table 1. The qRT-PCR was conducted following previously reported methods [25,26]. Relative quantification of each gene was performed by the 2^−ΔΔCt^ method using beta-actin (*Actb*) for the MBH and liver, and ribosomal protein S18 (*Rps18*) for the iWAT as an internal control.

### 4.6. Blood Biochemical Analysis

Blood biochemicals were analyzed at the experimental end point following previously reported methods [25,26]. Briefly, the GLUCOCARD G+ meter was used to measure glucose content (Arkray). NEFA C-Test Wako (Wako Pure Chemical Industries, Osaka, Japan) was used to measure FFA levels. Triglyceride E-Test Wako (Wako Pure Chemical Industries) was used to measure TG levels. The Rebis Insulin-mouse T ELISA kit (Shibayagi) was used to measure insulin levels.

### 4.7. Statistical Analysis

Group differences between AAV-NPGL- and AAV-CTL-injected animals were assessed using the unpaired two-tailed Student’s *t*-test and Mann–Whitney *U* test. *p* values < 0.05 were considered significant. Statistical comparisons between every two groups at each time point were conducted with the unpaired two-tailed Student’s *t*-test in Figure 1B and the results of OGTT and IPITT.

## Figures and Tables

**Table 1:** Sequences of oligonucleotide primers for quantitative RT-PCR.

Gene	Sense Primer (5′ to 3′)	Antisense Primer (5′ to 3′)
*Npgl*	GGAACCATGGCTTAGGAAGG	TCTAAGGAGCTGAGAATATGCA
*Npy*	TATCTCTGCTCGTGTGTTTG	GATTGATGTAGTGTCGCAGA
*Agrp*	TGTTCCCAGAGTTCCCAGGTC	GCATTGAAGAAGCGGCAGTAGCAC
*Pomc*	AGCTGCCTTTCCGCGACA	ATCTATGGAGGTCTGAAGCA
*Gal*	GAGCCTTGATCCTGCACTGA	AGTGGCTGACAGGGTCACAA
*Acc*	TCCGCACTGACTGTAACCACAT	TGCTCCGCACAGATTCTTCA
*Fas*	AGGGGTCGACCTGGTCCTCA	GCCATGCCCAGAGGGTGGTT
*Chrebp* *α*	CGACACTCACCCACCTCTTC	TTGTTCAGCCGGATCTTGTC
*Cpt1a*	CCTGGGCATGATTGCAAAG	GGACGCCACTCACGATGTT
*Atgl*	AACACCAGCATCCAGTTCAA	GGTTCAGTAGGCCATTCCTC
*Hsl*	GCTGGGCTGTCAAGCACTGT	GTAACTGGGTAGGCTGCCAT
*Gapdh*	AAGGTCATCCCAGAGCTGAA	CTGCTTCACCACCTTCTTGA
*Slc2a4*	GTAACTTCATTGTCGGCATGG	AGCTGAGATCTGGTCAAACG
*Cd36*	TCCTCTGACATTTGCAGGTCTATC	AAAGGCATTGGCTGGAAGAA
*Pgc1* *α*	GCAACATGCTCAAGCCAAAC	TGCAGTTCCAGAGAGTTCCA
*Ucp1*	CAAAAACAGAAGGATTGCCGAAA	TCTTGGACTGAGTCGTAGAGG
*Dio2*	CCACCTTCTTGACTTTGCCA	GGTGAGCCTCATCAATGTATAC
*G6pase*	ACTGTGGGCATCAATCTCCTC	CGGGACAGACAGACGTTCAGC
*Pepck*	GTGCTGGAGTGGATGTTCGG	CTGGCTGATTCTCTGTTTCAGG
*Slc2a2*	GGCTAATTTCAGGACTGGTT	TTTCTTTGCCCTGACTTCCT
*Fgf21*	CCTCTAGGTTTCTTTGCCAACAG	AAGCTGCAGGCCTCAGGAT
*Actb*	GGCACCACACCTTCTACAAT	AGGTCTCAAACATGATCTGG
*Rps18*	CCTGAGAAGTTCCAGCACAT	TTCTCCAGCCCTCTTGGTG

## Data Availability

No big data repositories needed. The raw data supporting the findings of this manuscript will be made available by the corresponding author, K.U., to any qualified researchers upon reasonable request.

## Supplementary Materials

Supplementary materials can be found at https://www.mdpi.com/article/10.3390/ijms22094681/s1.
