# Supplementary material for: Effects of Overexpression of Neurosecretory Protein GL-Precursor Gene on Glucose Homeostasis and Insulin Sensitivity in Mice
Source: Int J Mol Sci. 2021 Apr 28;22(9):4681. doi: 10.3390/ijms22094681 (PMC8125475; doi:10.3390/ijms22094681)
Supplement: Supplementary file 1 [file ijms-22-04681-s001.zip › ijms-1180272-supplementary(1).pdf]

Supplementary Materials

Effects of Overexpression of Neurosecretory Protein GL-Precursor Gene on Glucose Homeostasis and Insulin Sensitivity in Mice

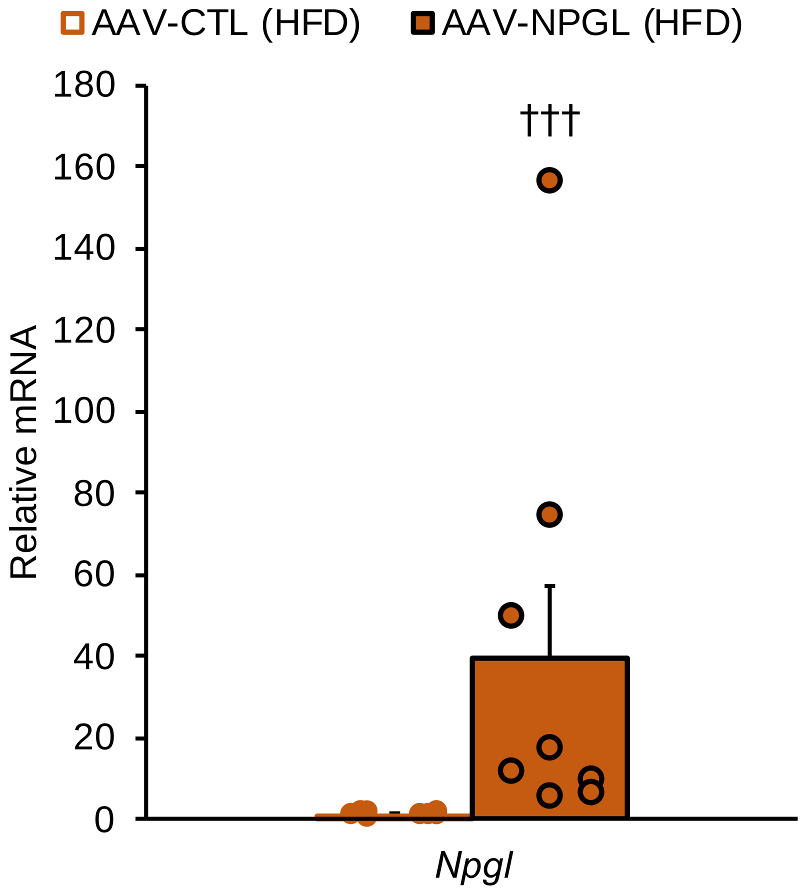

Figure S1. Efficacy of *Npgl* overexpression in the mediobasal hypothalamus at the experimental endpoint. The panel shows mRNA expression level of *Npgl* obtained upon injection the AAV-based control vector (AAV-CTL) or the AAV-based NPGL-precursor gene vector (AAV-NPGL) in high-fat diet (HFD)-fed mice. Circles represent individual data points. Each value represents the mean ± standard error of the mean ($n = 8$; †††$p < 0.005$ for Mann-Whitney U test).

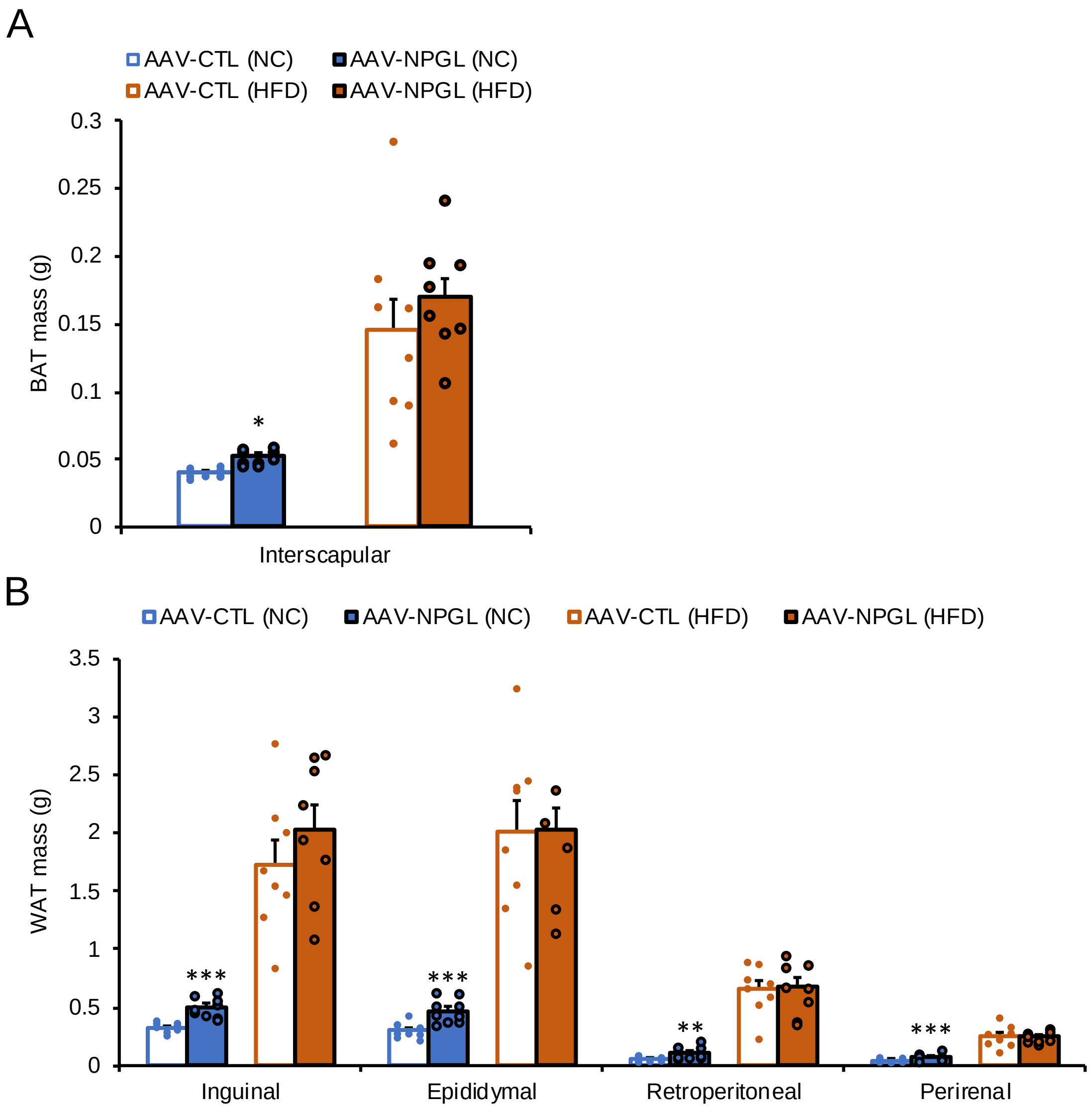

Figure S2. Effects of *Npgl* overexpression on fat accumulation in normal chow (NC)-fed and high-fat diet (HFD)-fed mice. The panels show the data obtained upon injection of the AAV-based control vector (AAV-CTL) or the AAV-based NPGL-precursor gene vector (AAV-NPGL) in NC-fed and HFD-fed mice. (**A**) Brown adipose tissue (BAT) mass. (**B**) Masses of inguinal, epididymal, retroperitoneal, and perirenal white adipose tissue (WAT). Circles represent individual data points. Each value represents the mean ± standard error of the mean (NC-fed mice: $n = 9$; *$p < 0.05$, **$p < 0.01$, ***$p < 0.005$ for Student's t-test, HFD-fed mice: $n = 8$).

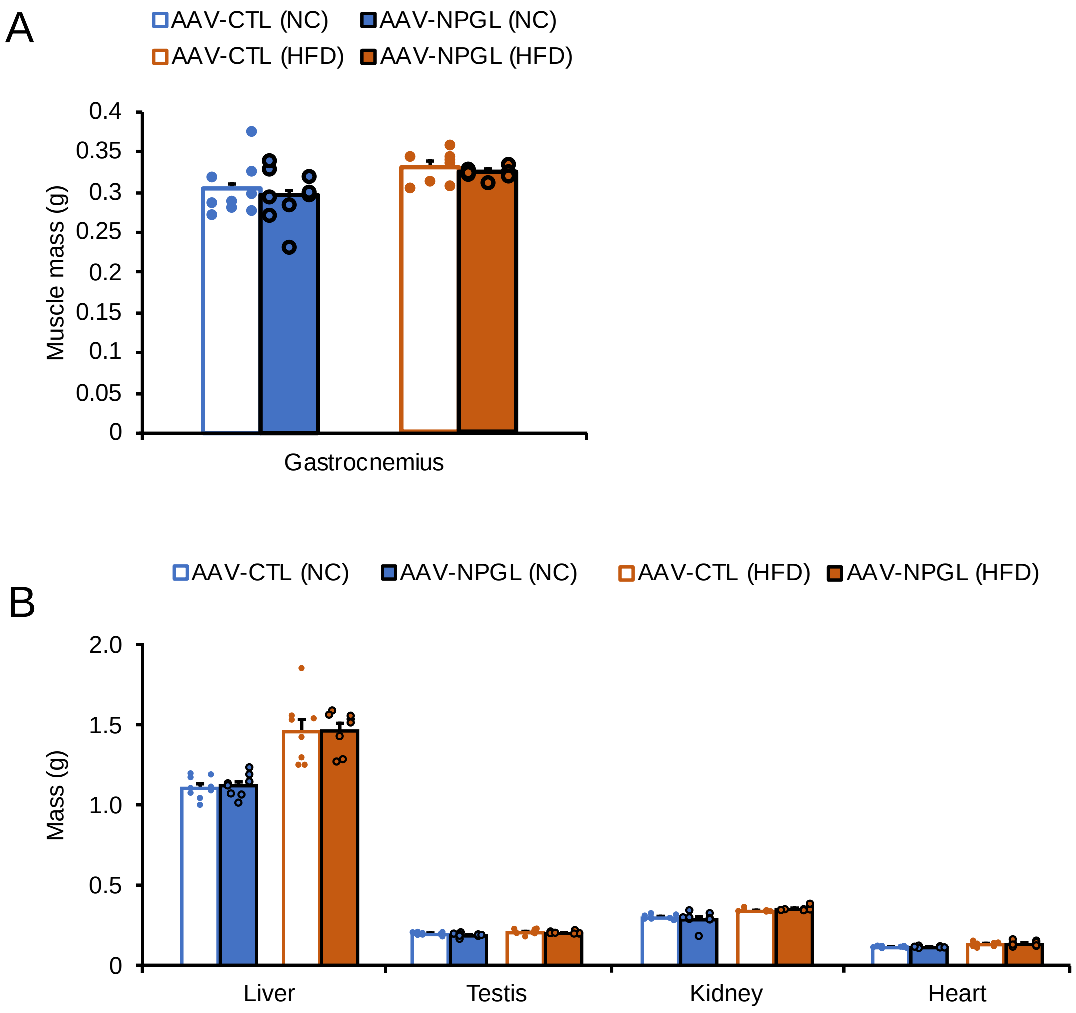

Figure S3. Effects of *Npgl* overexpression on muscle and organ masses in normal chow (NC)-fed and high-fat diet (HFD)-fed mice. The panels show the data obtained upon injection of the AAV-based control vector (AAV-CTL) or the AAV-based NPGL-precursor gene vector (AAV-NPGL) in NC-fed and HFD-fed mice. (**A**) Gastrocnemius muscle mass. (**B**) Masses of the liver, testis, kidney, and heart. Circles represent individual data points. Each value represents the mean ± standard error of the mean (NC-fed mice: $n = 9$, HFD-fed mice: $n = 8$).

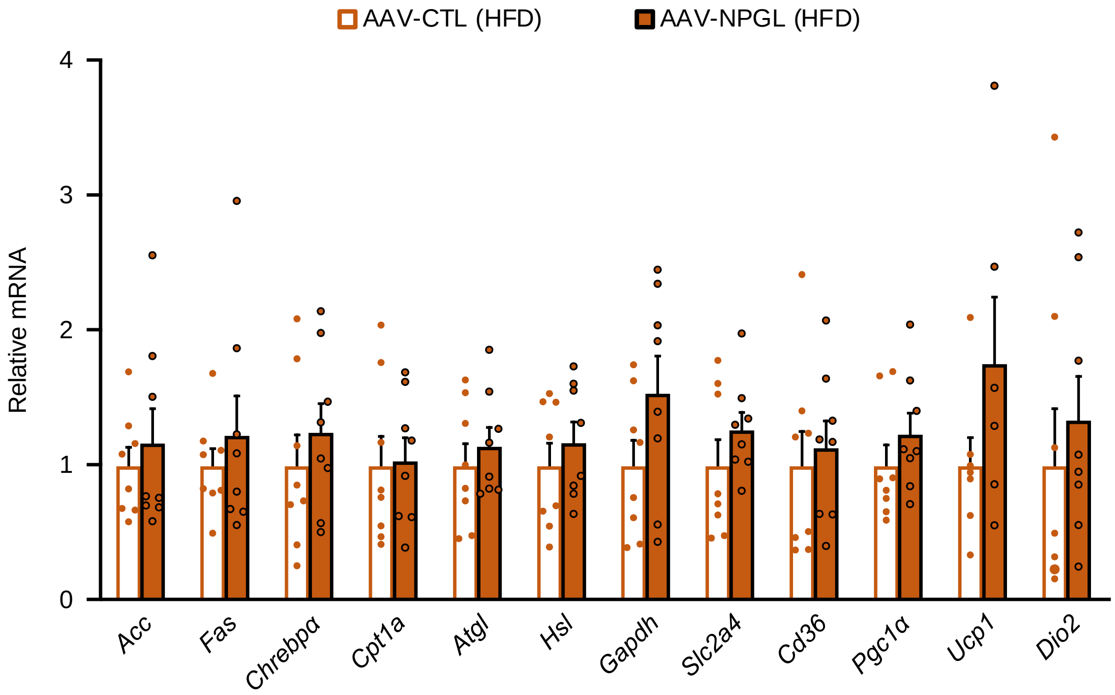

Figure S4. Effects of *Npgl* overexpression on mRNA expression of genes related to lipid metabolism, glycolysis, glucose and lipid uptake, and browning in the inguinal white adipose tissue of high-fat diet (HFD)-fed mice. The graph shows the data obtained upon injection of the AAV-based control vector (AAV-CTL) or the AAV-based NPGL-precursor gene vector (AAV-NPGL) in HFD-fed mice. mRNA expression levels of acetyl-CoA carboxylase (*Acc*), fatty acid synthase (*Fas*), carbohydrate-responsive element-binding protein α (*Chrebpα*), carnitine palmitoyltransferase 1a (*Cpt1a*), adipose triglyceride lipase (*Atgl*), hormone-sensitive lipase (*Hsl*), glyceraldehyde-3-phosphate dehydrogenase (*Gapdh*), solute carrier family 2 member 4 (*Slc2a4*), cluster of differentiation 36 (*Cd36*), peroxisome proliferator-activated receptor γ coactivator 1α (*Pgc1α*), uncoupling protein 1 (*Ucp1*), and type II iodothyronine deiodinase (*Dio2*). Circles represent individual data points. Each value represents the mean ± standard error of the mean (n = 6–8).

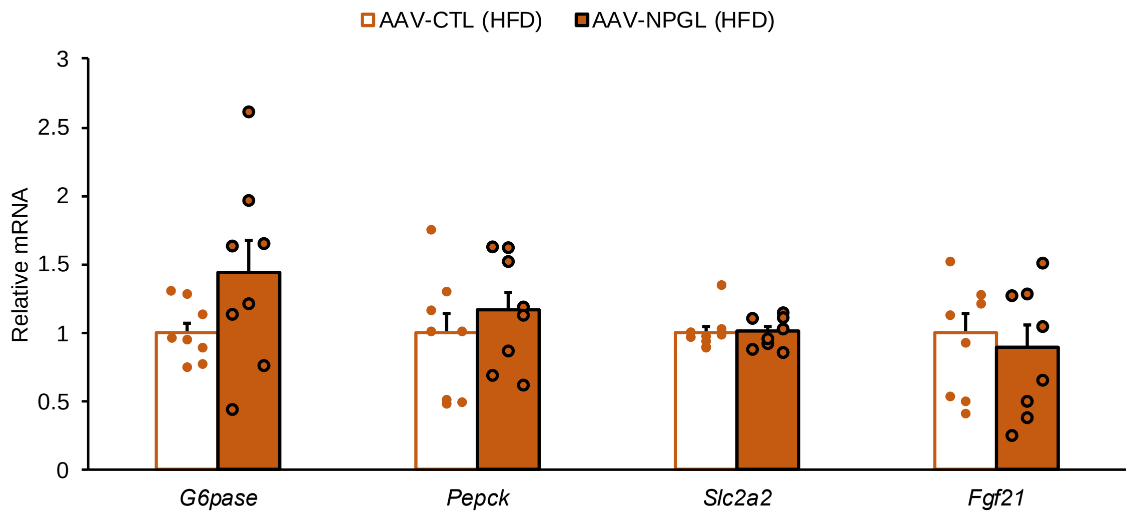

Figure S5. Effects of *Npgl* overexpression on mRNA expression of genes related to gluconeogenesis and glucose uptake in the liver of high-fat diet (HFD)-fed mice. The graph shows the data obtained upon injection of the AAV-based control vector (AAV-CTL) or the AAV-based NPGL-precursor gene vector (AAV-NPGL) in HFD-fed mice. mRNA expression levels of glucose-6-phosphatase (*G6pase*), phosphoenolpyruvate carboxykinase (*Pepck*), solute carrier family 2 member 2 (*Slc2a2*), and fibroblast growth factor 21 (*Fgf21*). Circles represent individual data points. Each value represents the mean ± standard error of the mean (n = 8).
